# Modified streptozotocin‐induced diabetic model in rodents

**DOI:** 10.1002/ame2.12497

**Published:** 2024-09-30

**Authors:** Anton Lennikov, Farris ElZaridi, Menglu Yang

**Affiliations:** ^1^ Department of Ophthalmology, Schepens Eye Research Institute, Mass Eye and Ear Harvard Medical School Boston Massachusetts USA

**Keywords:** hyperglycemia, non‐fasting, streptozotocin, Type 1 diabetes mellitus

## Abstract

Streptozotocin (STZ)‐induced type I diabetes mellitus (DM) models have been pivotal in diabetes research due to their ability to mimic the insulin‐dependent hyperglycemia akin to human type I diabetes. However, these models often suffer from poor induction rates and low survival post‐STZ induction, especially in long‐term experiments, necessitating insulin supplementation, which introduces additional variables to experiments. To address this, we present a novel modification to the STZ‐induced DM model in C57BL/6J mice to improve survival rates without insulin supplementation. Our method involves non‐fasting, low‐dose STZ injections dissolved in pH‐neutral phosphate buffer saline instead of acidic sodium citrate buffer, administered over 5 days. We observed hyperglycemia induction in 94.28% of mice within a week post‐injection, with stable high blood glucose levels, stable body weight, and minimal mortality up to 21 weeks. Notably, omitting 10% sucrose in water and fasting did not affect hyperglycemia induction. Our findings suggest that the modified protocol not only decreases the experimental effort of the researchers, but reduces animal stress and mortality, thus enhancing experimental outcomes and animal welfare. By optimizing the STZ‐induced DM model in C57BL/6J mice, our study provides a valuable resource for researchers aiming to study diabetes and its complications while minimizing experimental variability and animal usage.

## Graphical abstract

Streptozotocin (STZ) induced type I diabetes mellitus (DM) models have been pivotal in diabetes research due to their ability to mimic the insulin‐dependent hyperglycemia akin to human type‐I diabetes. However, these models often suffer from poor induction rates and low survival post‐STZ injection, especially in long‐term experiments, necessitating insulin supplementation, which introduces additional variables to experiments. We present a novel modification to the STZ‐induced DM model in C57BL6J mice to improve survival rates without insulin supplementation. Our method involves non‐fasting, low‐dose STZ injections dissolved in pH neutral PBS instead of acidic sodium citrate buffer, administered over 5 days. We observed hyperglycemia induction in 94.28% of mice within a week post‐injection, with stable high blood glucose levels, stable body weight, and minimal mortality up to 21 weeks.

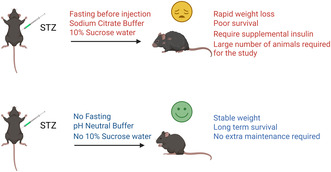

## 1 INTRODUCTION

Streptozotocin (STZ) is a naturally occurring alkylating substance with selective toxicity to the pancreatic β cells due to STZ's structural similarity to glucose and its ability to utilize the GLUT2 transport protein in pancreatic β cells.^1^, ^2^ STZ‐induced type I diabetes mellitus (DM) models have been developed in rodents for decades by depleting the insulin‐producing β‐cells in the pancreas, thereby causing hyperglycemia with a low insulin level.^1^, ^3^ This inducible model can be established in genetically modified animals for mechanistic analysis and, therefore, has been widely used in a variety of research fields, including diabetes and diabetic complications, such as neuropathy, nephropathy, cardiomyopathy, and retinopathy.

Over the years, different groups have reported multiple STZ administration variations, including single high‐dose bolus injection^4^ and multiple‐dose low‐dose injection,^2^ with or without fasting^5^ or in combination with a high‐fat diet.^6^ Chaudhry et al. for instance, previously reported that animal fasting has no significant influence on STZ model induction and course.

According to the literature, the basic procedures include^7^, ^8^:

1. Fasting the mice 4–6 h before STZ treatment.
2. Dissolving STZ in sodium citrate buffer with a pH of 4.5 to improve the stability of the solution.^7^
3. Daily intraperitoneal (i.p.) injection of 30–50 mg/kg of STZ for 5 days.^9^, ^10^
4. Supplemental 10% sucrose in the animal drinking water during the days of injection.^4^

However, a major concern of these STZ‐induced models is the low survival rate of 20 weeks following the STZ injection.^7^ This undesirable loss of study subjects during observation results in increased experimental animal use, extra spending, and a shortened observation window, which raises a concern with studies of complications during the later stage of disease progress. To address this issue, insulin is required to rescue the mice from hyperglycemia.^11^ However, as insulin use leads to additional metabolic and mitogenic effects, introducing insulin may add undesirable variables to the experimental design.

Over the course of years of practical application, researchers have modified the procedure to increase the survival rate.^5^, ^12^ Herein, we introduce a novel modification of the STZ‐induced diabetes model in the C57BL/6J strain to increase the survival rate without needing insulin application while reducing the overall effort in the model induction.

## 2 METHODS

### 2.1 Animals and reagents

All animals were housed at a specific‐pathogen‐free animal facility at Schepes Eye Research Institute. All animal experiments were approved by the Institutional Animal Care and Use Committee of the Schepens Eye Research Institute and were conducted in compliance with the guidelines of the ARVO (Association for Research in Vision and Ophthalmology, Rockville, MD, USA).

C57BL/6J male mice, 6–8 weeks old (Jackson Laboratory, Bar Harbor, ME, USA), with an initial weight of 22 ± 2 g were used. Mice were caged at 24°C, with a 12 h light–dark cycle, and always had free access to normal rodent diet and water.

The animals were fed with PicoLab Rodent Diet 20 (3005740–220, PicoLab, Durham, NC, USA). The diet provided calories (3 kcal/g) in the form of protein (24.495%), fat (13.122%) and carbohydrates (62.382%).

The reagents used were: sterile PBS (pH = 7.4); fresh STZ (Sigma, MO, USA), stored at −20°C; 1.5 mL sterile Eppendorf tubes,and 1 mL sterile syringe with 25 G needles.

### 2.2 Preparation

The mice were weighed and the amount of STZ required for a dose of 40 mg/kg of body weight was calculated. Appropriate personal protective equipment should be worn when handling STZ. STZ was removed from −20°C freezer, weighed to provide the calculated amount, and transferred into Eppendorf tubes. Doses of 40 mg/kg/mouse were prepared based on the animals' weights (i.e., a 30 g mouse requires 1.2 mg STZ). The amount required for the number of mice was weighed (i.e., for 5 mice, 1.2 mg/mouse × 5 mice = 6 mg). The volume of PBS required to give 100 μL/mouse (i.e., 500 μL for 5 mice) was prepared. Do not mix PBS with STZ at this stage. STZ was dissolved with PBS immediately before injecting the mice; the injections were given *within 5 min*.

Using a 1 mL syringe with a 25 G needle, the STZ solution was injected i.p. at 40 mg/kg body weight. For the control group, an equal amount of sterile PBS was injected i.p.

The mice were returned to their disposable cages with free access to normal food and water. Water gel for additional animal hydration (ClearH2O, Westbrook, ME, USA) was optionally provided for each cage. The injection protocol was repeated for the next 4 days, making the STZ treatment period 5 days in total.

### 2.3 Post‐injection care

Blood glucose was monitored using one drop of blood sampled from the tail with a scalpel or a 23G needle blade at the baseline. Blood glucose was then measured weekly to validate the hyperglycemia induction. Off‐the‐counter glucometers are reported to be suitable for mouse blood glucose measurements^13^; however, because these instruments are designed for human use, the maximum glucose readings are frequently capped at 600 mg/dL. To address this issue, the blood sample can be diluted with 0.9% saline before measurements, or a dedicated veterinary glucometer can be used.

Body weights were monitored at the baseline and then weekly after STZ injection.

## 3 RESULTS

**FIGURE 1:**
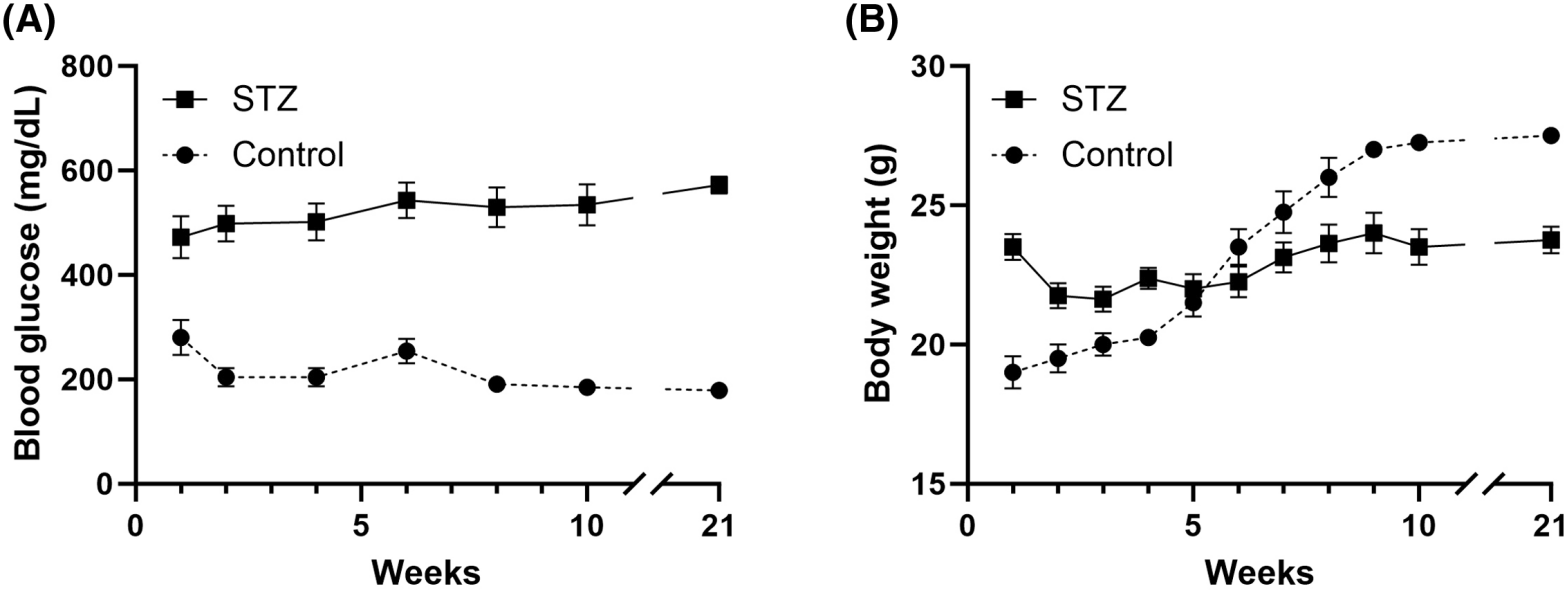
Blood glucose levels (A) and body weight (B) in streptozotocin (STZ)‐treated (indicated by the black squares) and control (indicated by the black circles) mice over 21 weeks of observations. Mice with blood glucose levels above 350 mg/dL were considered diabetic. STZ, *n* = 31; Control, *n* = 8.

Of the 35 mice subjected to this procedure, 33 developed hyperglycemia within 1 week post‐injection. The blood glucose in the STZ group continued to increase during weeks 2–6 after injection and stabilized after week 6 (Figure 1A). All mice exhibited a slight weight loss over the first 2 weeks after STZ injection, then gradually gained weight throughout the observation, stabilizing at week 7 (Figure 1B). Two of the mice were excluded from the cohort before the desired study endpoint due to significant weight loss greater than 20% from the baseline at weeks 5 and 7, respectively, making the survival rate of this procedure 94.28%. We observed the animals for up to 21 weeks after STZ injection without the need for re‐injection or any signs of severe complications.

## 4 DISCUSSION

Here, we present our recent findings in a new non‐fasting low‐dose STZ inducible type I DM mouse model. We have made several adjustments to the classic procedure, including: (1) no fasting before the STZ injection; (2) normal drinking water instead of 10% sucrose water during the 5‐day induction of the model; (3) dissolving STZ in sterile PBS with a neutral pH instead of sodium citrate buffer.

The results indicate successful induction of hyperglycemia and minimal mortality. The reduced lethal complications allow us to reduce the number of animals used in the experimental design, leading to reduced experimental effort and improved animal welfare.

The redundancy of fasting has already been discussed by Chaudhry et al. who showed that STZ is equally diabetogenic whether administered to fed or fasted mice, which we confirmed in our experiments.^5^

According to the literature, administration of 10% sucrose water is intended to rescue animals from fatal hypoglycemia during the initial STZ injection, which is associated with the uncontrolled release of insulin from dying β‐cells.^4^ Because we observed no hypoglycemia symptoms during the STZ injection phase, we omitted the sucrose water in the current settings. We speculate that the low stress level of our animals is due to the lack of fasting and replacing the acidic sodium citrate buffer with neutral PBS, which reduced irritability in the visceral organs of the peritoneum. The pH of citrate buffer for STZ injections is 4.0 intended to improve the stability of dissolved STZ. A pH of 4.0 is acidic and outside of the physiologic pH range of 7.3–7.4 for mouse i.p. administration. Administering substances outside the recommended pH ranges can lead to tissue necrosis and vascular thrombosis.^14^ The feasibility of dissolving STZ in a pH‐neutral solution has been described by Deeds et al.^15^ and our high success rate in inducing hyperglycemia provided additional evidence for their point. For the current study, we did not include a classic STZ injection control to reduce the number of animals. The mortality rate of the classic STZ model has been reported as greater than 20%^4^; the mortality rate using our method is markedly lower than the classical method. Future investigations will test the current method in females and other strains of mice.

Our observations have several limitations. (1) We only omitted 10% sucrose water based on observations of the animal stress level, without frequently monitoring blood glucose. In theory, STZ injection would cause transient hypoglycemia in mice. However, according to our observation, our STZ induction method does not cause apparent animal stress or death. (2) The effectiveness of our modified simplified STZ diabetes model—omitting citrate buffer, fasting, and including supplemental glucose—was evaluated primarily based on weight, glucose levels, and mortality rates. We did not investigate changes at the protein and transcriptome levels in DM target organs such as the retina or kidneys to compare with traditional STZ model induction schedules. Therefore, further studies are necessary to understand the potential impact of these modifications.

In summary, we have described a modified method to induce type I DM in C57BL/6J mice that requires less effort and presents an excellent induction rate, stable high glucose, and long‐term survival rates of diabetic animals.

## AUTHOR CONTRIBUTIONS

**Farris ElZaridi:** Data curation; formal analysis; investigation; writing – review and editing. **Anton Lennikov:** Conceptualization; data curation; investigation; methodology; writing – original draft. **Menglu Yang:** Conceptualization; investigation; project administration; supervision; funding acquisition; validation; writing – original draft.

## FUNDING INFORMATION

This research is funded by the Massachusetts Lions Charitable Foundation (MLY) and Department of Defense (USA) HT9425‐23‐1‐1045 (AL).

## CONFLICT OF INTEREST STATEMENT

The authors declare that the research was conducted in the absence of any commercial or financial relationships that could be construed as a potential conflict of interest.

## ETHICS STATEMENT

All animal experiments were approved by the Institutional Animal Care and Use Committee of the Schepens Eye Research Institute and were conducted in compliance with the guidelines of the ARVO (Association for Research in Vision and Ophthalmology).

## Data Availability

All manuscript data is incorporated in the manuscript text.
